# The effect of white matter hyperintensities on statistical analysis of diffusion tensor imaging in cognitively healthy elderly and prodromal Alzheimer's disease

**DOI:** 10.1371/journal.pone.0185239

**Published:** 2017-09-21

**Authors:** Daniel Svärd, Markus Nilsson, Björn Lampinen, Jimmy Lätt, Pia C. Sundgren, Erik Stomrud, Lennart Minthon, Oskar Hansson, Danielle van Westen

**Affiliations:** 1 Diagnostic Radiology, Lund University, Lund, Sweden; 2 Medical Imaging and Physiology, Skåne University Hospital, Lund, Sweden; 3 Lund University Bioimaging Center, Lund University, Lund, Sweden; 4 Medical Radiation Physics, Lund University, Lund, Sweden; 5 Clinical Memory Research, Lund University, Malmoö, Sweden; 6 Memory Clinic, Skåne University Hospital, Lund, Sweden; Brainnetome Center & The National Laboratory of Pattern Recognition, CHINA

## Abstract

Diffusion tensor imaging (DTI) has been used to study microstructural white matter alterations in a variety of conditions including normal aging and Alzheimer's disease (AD). White matter hyperintensities (WMH) are common in cognitively healthy elderly as well as in AD and exhibit elevated mean diffusivity (MD) and reduced fractional anisotropy (FA). However, the effect of WMH on statistical analysis of DTI estimates has not been thoroughly studied. In the present study we address this in two ways. First, we investigate the effect of WMH on MD and FA in the dorsal and ventral cingulum, the superior longitudinal fasciculus, and the corticospinal tract, by comparing two matched groups of cognitively healthy elderly (*n* = 21 + 21) with unequal WMH load. Second, we assess the effects of adjusting for WMH load when comparing MD and FA in prodromal AD subjects (*n* = 83) to cognitively healthy elderly (*n* = 132) in the abovementioned white matter tracts. Results showed the WMH in cognitively healthy elderly to have a generally large effect on DTI estimates (Cohen’s *d* = 0.63 to 1.27 for significant differences in MD and −1.06 to −0.69 for FA). These effect sizes were comparable to those of various neurological and psychiatric diseases (Cohen’s *d* = 0.57 to 2.20 for differences in MD and −1.76 to −0.61 for FA). Adjusting for WMH when comparing DTI estimates in prodromal AD subjects to cognitively healthy elderly improved the explanatory power as well as the outcome of the analysis, indicating that some of the differences in MD and FA were largely driven by unequal WMH load between the groups rather than alterations in normal-appearing white matter (NAWM). Thus, our findings suggest that if the purpose of a study is to compare alterations in NAWM between two groups using DTI it may be necessary to adjust the statistical analysis for WMH.

## Introduction

Diffusion-weighted MRI is a non-invasive technique capable of detecting microstructural tissue alterations such as axonal loss and demyelination in the human brain *in vivo* [1–3]. As such, its popularity has been growing steadily during the last two decades. Diffusion tensor imaging (DTI) yields estimates of e.g. the mean diffusivity (MD) and the fractional anisotropy (FA), and has been used to study human brain development and normal aging as well as a wide variety of neurological and psychiatric conditions [4–8].

White matter hyperintensities (WMH) are, compared to normal-appearing white matter (NAWM), visualized as hyperintense regions in the white matter on T2-weighted MRI and are most commonly thought to be due to small vessel disease [9–11]. They are typically located in periventricular or deep cortical regions and are histopathologically characterized by ischemic changes and a varying degree of gliosis, axonal loss, and demyelination. WMH are interchangeably called white matter lesions, white matter changes, and leukoaraiosis amongst others [11–13]. These changes are relatively common in cognitively healthy elderly with the prevalence varying substantially between different studies [14–16]. Some studies report that more than 90% of individuals older than 60 years exhibit WMH [17].

DTI is sensitive to WMH, exhibiting elevated MD and reduced FA in affected white matter regions [18–20]. However, the relationship between alterations in NAWM, detectable only with DTI, and WMH, also detectable with T2-weighted MRI, has only recently been investigated more thoroughly [21,22]. It has been proposed that WMH constitute a histopathological continuum of varying degree of white matter pathology [23]. Several recent studies have also investigated the effect of WMH on DTI estimates in normal aging and concluded that WMH only partially explain the elevated MD and reduced FA seen in normal aging, suggesting that alterations in NAWM as quantified with DTI may represent an early phase and WMH the late phase of the same common pathophysiological phenomenon [24,25].

Given that WMH are seen in normal aging, it is reasonable to suspect that the mere presence of WMH in cognitively healthy elderly are associated to elevated MD and reduced FA. Two recent studies investigated this and observed that subjects having WMH were more likely to have elevated MD and reduced FA in general compared to healthy subjects not having WMH [26,27].

Due to the association between DTI estimates and the presence of WMH, unequal WMH load between two groups may confound group comparisons, and lead to differences being partially due to the difference in WMH load rather than more disease-specific effects of alterations in NAWM. However, this methodological issue is not always considered and accounted for in DTI research, and the magnitude of the effect of WMH on DTI estimates in healthy subjects has not yet been fully investigated. The two above referenced studies included only a small number of subjects with WMH (*n* = 12 and *n* = 8, respectively) [26,27]. Also, most of the subjects were in the fourth to fifth decades of life, wherefore it is uncertain to what degree the results apply to subjects in the sixth to eight decades of life, which are known to have a higher prevalence of WMH. Moreover, to our knowledge, no previous study has evaluated how the level of WMH load in a control group affects the comparison of DTI estimates to a diseased group.

Amnestic mild cognitive impairment (aMCI) is characterized by cognitive impairment greater than expected for a particular age and educational level, but not significant enough to interfere with activities of daily life or fulfilling the criteria for dementia [28]. Although aMCI is etiologically a heterogeneous entity, in a majority of cases aMCI is an expression of prodromal Alzheimer's disease (AD) and it has been shown that the ratio between Aβ_42_ and Aβ_40_ in cerebrospinal fluid (CSF) predicts conversion to AD rather well [29]. aMCI and AD are both associated with alterations in NAWM that are thought to be disease-specific, as quantified with DTI, but they are also associated with elevated levels of WMH [5,30]. Thus, it is possible that including controls with high WMH load in a study of prodromal AD subjects versus cognitively healthy elderly may lead to improper conclusions about disease-specific alterations in NAWM detectable only with DTI in the prodromal AD group. That is, inclusion of controls with high WMH load may lead to false negative results. On the other hand, inclusion of controls with low WMH load in a study of prodromal AD subjects with high WMH load may lead to an overestimation of disease-specific alterations in NAWM.

We therefore hypothesized that WMH can affect the results of a statistical analysis in DTI when there is a difference in WMH load between the investigated groups. The aim of this study was thus to, first, study the effect of unequal WMH load between two matched groups of cognitively healthy elderly on DTI estimates in several major white matter tracts and, second, to assess the effect of WMH when comparing prodromal AD subjects to cognitively healthy elderly. This aim was addressed in two ways. First, we compared MD and FA in the right and left dorsal and ventral cingulum, the right and left superior longitudinal fasciculus (SLF), and the right and left corticospinal tract (CST) in cognitively healthy elderly with different levels of WMH load and estimated the effect size of WMH on DTI estimates. Second, we compared MD and FA in the abovementioned white matter tracts between prodromal AD subjects and cognitively healthy elderly in two analyses, unadjusted and adjusted for WMH, respectively. The abovementioned tracts were selected because the dorsal and ventral cingulum and the SLF, but not the CST, is associated with elevated MD and reduced FA in AD subjects, and the dorsal cingulum and the SLF, but not the ventral cingulum and the CST, are at least partially located in areas were WMH are common [5,17,30,31]. This selection thereby allowed us to more carefully disentangle the effect of unequal WMH load between groups on DTI estimates from more disease-specific alterations in NAWM in prodromal AD.

## Methods

### Ethics statement

The study was approved by the local Ethics Committee at Lund University and conducted according to the provisions of the Helsinki Declaration. Written informed consent was obtained from all subjects.

### Study population

Cognitively healthy elderly (*n* = 132; mean age 71.6±4.5 years; 44.7% males) with a non-pathological Aβ_42_/Aβ_40_ ratio (i.e. Aβ_42_/Aβ_40_ > 0.1) were recruited from the Malmö Diet and Cancer Study, an epidemiological study part of the European Prospective Investigation into Diet and Cancer in the city of Malmö, Sweden (Table 1) [29,32]. Inclusion and exclusion criteria have been described previously [33]. Briefly, subjects were eligible for inclusion if they were above 60 years old, scored 27−30 points on Mini Mental State Examination (MMSE) at screening visit [34], did not suffer from any subjective cognitive impairment, and were fluent in Swedish. Exclusion criteria included presence of severe neurological disease (e.g. stroke, Parkinson’s disease or multiple sclerosis) or psychiatric disease (e.g. severe depression or psychotic syndromes).

**Table 1:** Sample characteristics of the study population.

	Cognitively healthy elderly	Cognitively healthy elderly with lower^a^ WMH load	Cognitively healthy elderly with higher^b^ WMH load	Prodromal AD subjects	*p* (Cognitively healthy elderly vs. prodromal AD subjects)
*n*	132	21	21	83	
Age (mean±SD)	71.6±4.5	72.6±3.8	74.0±4.9	71.4±5.4	0.800
Sex (% males)	44.7	52.4	47.6	53.0	0.237
CVD (% total)	47.7	76.2	61.9	40.7	0.322
MMSE (median)	29.3	29.2	29.1	26.9	< 0.001
Aβ_42_/Aβ_40_ (%; mean±SD)	0.14±0.02	0.14±0.02	0.14±0.02	0.07±0.02	< 0.001
VV (% ICV; mean±SD)	2.18±1.00	2.38±0.90	2.75±1.04	2.65±1.20	0.003
WMH volume (% ICV; mean±SD)	0.70±0.91	0.28±0.15	1.82±1.11	1.17±1.38	< 0.001

AD = Alzheimer's disease, CVD = cardiovascular disease, ICV = intracranial volume, MMSE = mini mental state examination, VV = ventricle volume, WMH = white matter hyperintensities.

^a^WMH volume ≤ 0.5% of the total intracranial volume, corresponding approximately to Fazekas score 0−1.

^b^WMH volume ≥ 1% of the total intracranial volume, corresponding approximately to Fazekas score 2−3.

aMCI subjects (*n* = 83; mean age 71.4±5.4 years; 53.0% males) with pathological Aβ_42_/Aβ_40_ ratio (i.e. Aβ_42_/Aβ_40_ ≤ 0.1) indicating high risk of conversion to AD were selected from the prospective and longitudinal Swedish BioFINDER study (Biomarkers For Identifying Neurodegenerative Disorders Early and Reliably; http://www.biofinder.se; Table 1) [29]. Subjects were classified as aMCI based on a neuropsychological battery of examinations and clinical assessments. They were enrolled consecutively at three memory outpatient clinics in Sweden and were assessed by physicians with special interest in dementia disorders. Subjects were eligible for inclusion if they were referred to the memory clinics due to cognitive impairment, presented objective memory impairment according to neuropsychological assessments, did not fulfill the criteria for dementia, scored 24−30 points on MMSE, were aged 60−80 years, and were fluent in Swedish. Exclusion criteria included cognitive impairment that without doubt could be explained by another condition (other than prodromal dementia), severe somatic disease, and refusing lumbar puncture or neuropsychological investigation.

### MRI acquisition and post-processing

Data acquisition was performed on a Siemens Trio 3 T MRI scanner using a standard 12-channel head coil. DTI data were acquired using a single-shot EPI sequence (TR/TE = 8200/86 ms/ms) with diffusion encoding in 64 directions using *b* values of 0 and 1000 s/mm^2^. In total, 60 contiguous axial slices with a spatial resolution of 2×2×2 mm^3^ were acquired. For the assessment of WMH load, 27 axial slices of T2-weighted FLAIR imaging were acquired (TR/TE/TI = 9000/89/2500 ms/ms/ms) at a spatial resolution of 0.7×0.7×5.2 mm^3^. The volumetric analysis was based on a coronal MPRAGE sequence (TR/TE/TI = 1950/3.37/900 ms/ms/ms), with a spatial resolution of 1.0×1.0×1.2 mm^3^ and a flip angle of 9°.

All DTI data were corrected for motion and eddy current induced artifacts by registering images to the first non-diffusion weighted image using Elastix [35,36]. Parameter maps of MD and FA were calculated from the diffusion tensor eigenvalues using in-house developed software implemented in Matlab (MATLAB 2013a, The MathWorks Inc., Natick, MA, USA). This software fitted the diffusion tensor using heteroscedasticity-corrected linear least squares regression. DTI volumes were registered to MNI152 standard-space using the registration algorithm of FLIRT and FNIRT, parts of the FMRIB Software Library [37,38].

Subjects that exhibited gross motion artifacts on DTI parameter maps or incidental findings such as meningioma, severe atrophy or old infarction were excluded from subsequent analysis.

### Tractography

**Fig 1:**
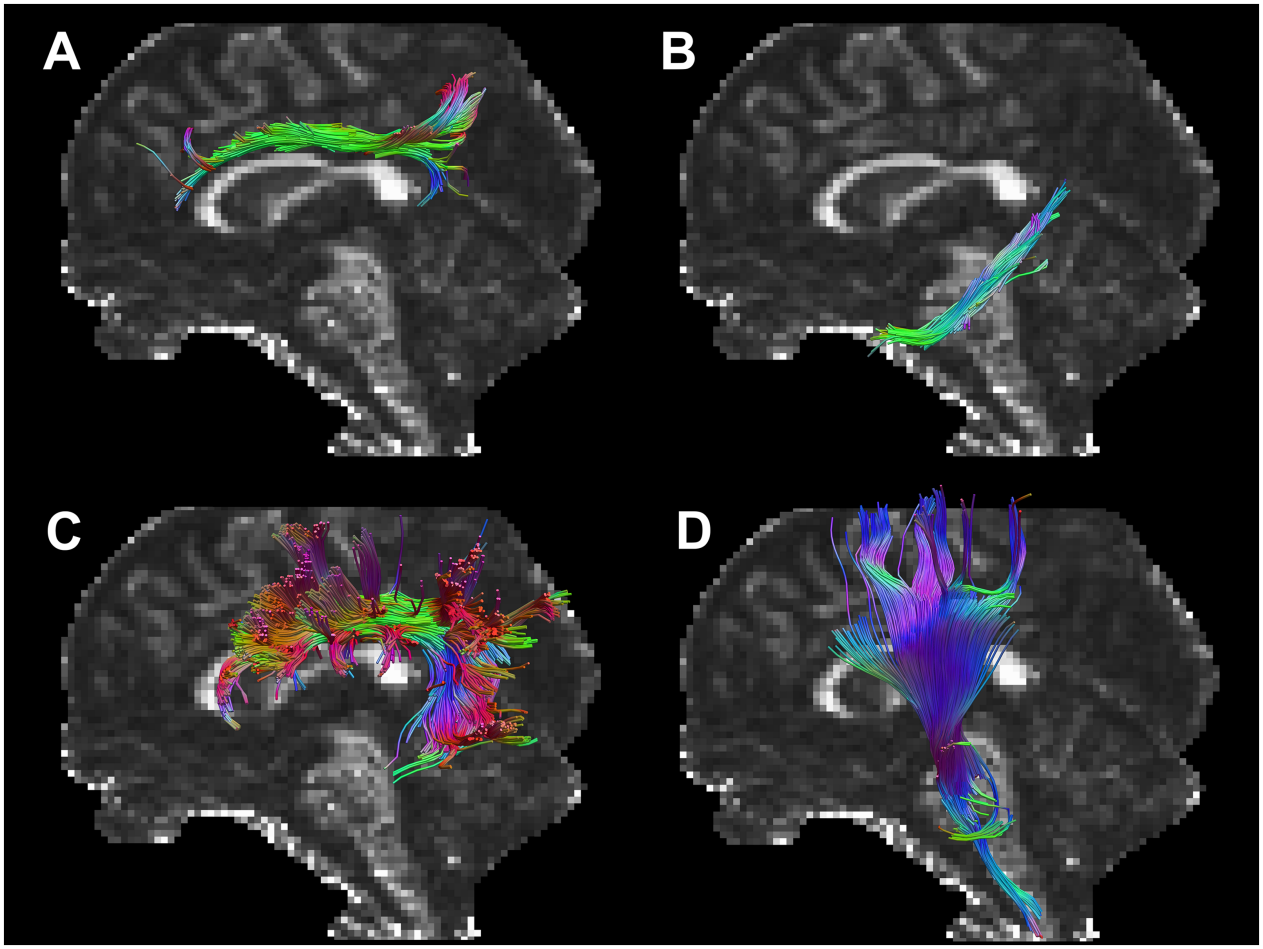
Graphical rendering of tractographies in a representative subject. Tractographies of the left-hand side of the dorsal cingulum (A), the ventral cingulum (B), the superior longitudinal fasciculus (SLF; C), and the corticospinal tract (CST; D) segmented from a whole-brain tractography and superimposed on a mid-sagittal FA map.

The investigated white matter tracts were generated using a semi-automated tractography method (Fig 1). The method utilized multiple regions of interest (ROIs) defined in MNI152 standard-space and warped to native-space using FNIRT results. The ROIs were then used to segment streamlines from a whole-brain tractography generated in TrackVis (thresholds were set to 0.2 for FA and 30° for the angle) [39]. This method was chosen over manual ROI definition in native-space, which would have required an inordinate analysis time due to the high number of subjects and white matter tracts. To include streamlines running along the whole white matter tract, as well as shorter streamlines in the same anatomical region, an ‘AND’-ROI covering the entire white matter tract was defined for each of the eight tracts in MNI152 standard-space, according to the ICBM-DTI-81 white matter labels atlas [40]. That is, the volume of the entire tract, as defined in the atlas, was projected from the atlas to standard-space and further to subject-space for each subject. To exclude streamlines from nearby white matter tracts intersecting the desired tract, a ‘NOT’-ROI was defined around their anatomical region of the tract according to the JHU WM tractography atlas [41]. Technically, the same procedure for creating the 'AND'-ROIs was used to create the 'NOT'-ROIs, except that before the ROIs were projected to subject-space they were automatically enlarged by approximately five voxels from the border of the ROI. Then the actual tract volume was subtracted resulting in a shell-like ROI, with a thickness that corresponded to the enlargement of the original ROI, that surrounded the tract volume as defined in the atlas. The procedure was performed using in-house developed software implemented in Matlab, utilizing the warp-fields generated by FNIRT to warp ROIs from standard-space to subject-space, and TrackVis for streamline management. For each subject, the segmented white matter tracts were visually inspected and, if necessary, adjusted in subject-space. This semi-automated method was found to be more robust because automated delineation of the ROIs was less biased by inter- and intra-individual anatomical variation or different position of the head in the MRI scanner than manual placement of ROIs [42]. The average value of MD and FA for each white matter tract was used in subsequent analysis.

### Volumetric assessment

White matter atrophy is associated with elevated MD and reduced FA [21]. To be able to adjust subsequent analysis for this confound, ventricle volume was normalized by intracranial volume and was used as a proxy for white matter atrophy. FreeSurfer was used to perform automated segmentation of the MPRAGE volumes to calculate ventricle volume (ml) and intracranial volume (ml) (Table 1) [43].

### Assessment of WMH load

Automated segmentation of WMH volume (ml) was performed with Lesion segmentation tool (LST as implemented in SPM8) using T2-weighted FLAIR imaging and the MPRAGE volumes. WMH volume was normalized by intracranial volume and was used to describe WMH load quantitatively (Table 1) [44]. In addition, WMH load was evaluated qualitatively by one trained operator using the Fazekas rating scale (grade 0–3) [12].

### Assessment of cardiovascular disease

Cardiovascular disease is associated with both WMH and elevated MD and reduced FA [45–47]. To be able to adjust subsequent analysis for this confound the presence of indirect markers of cardiovascular disease, diabetes, hypertension, ischemic heart disease, transient ischemic attack, and stroke [48] were used to classify each subject as either having or not having cardiovascular disease (Table 1).

### Definition of subgroups of cognitively healthy elderly with unequal WMH load

**Fig 2:**
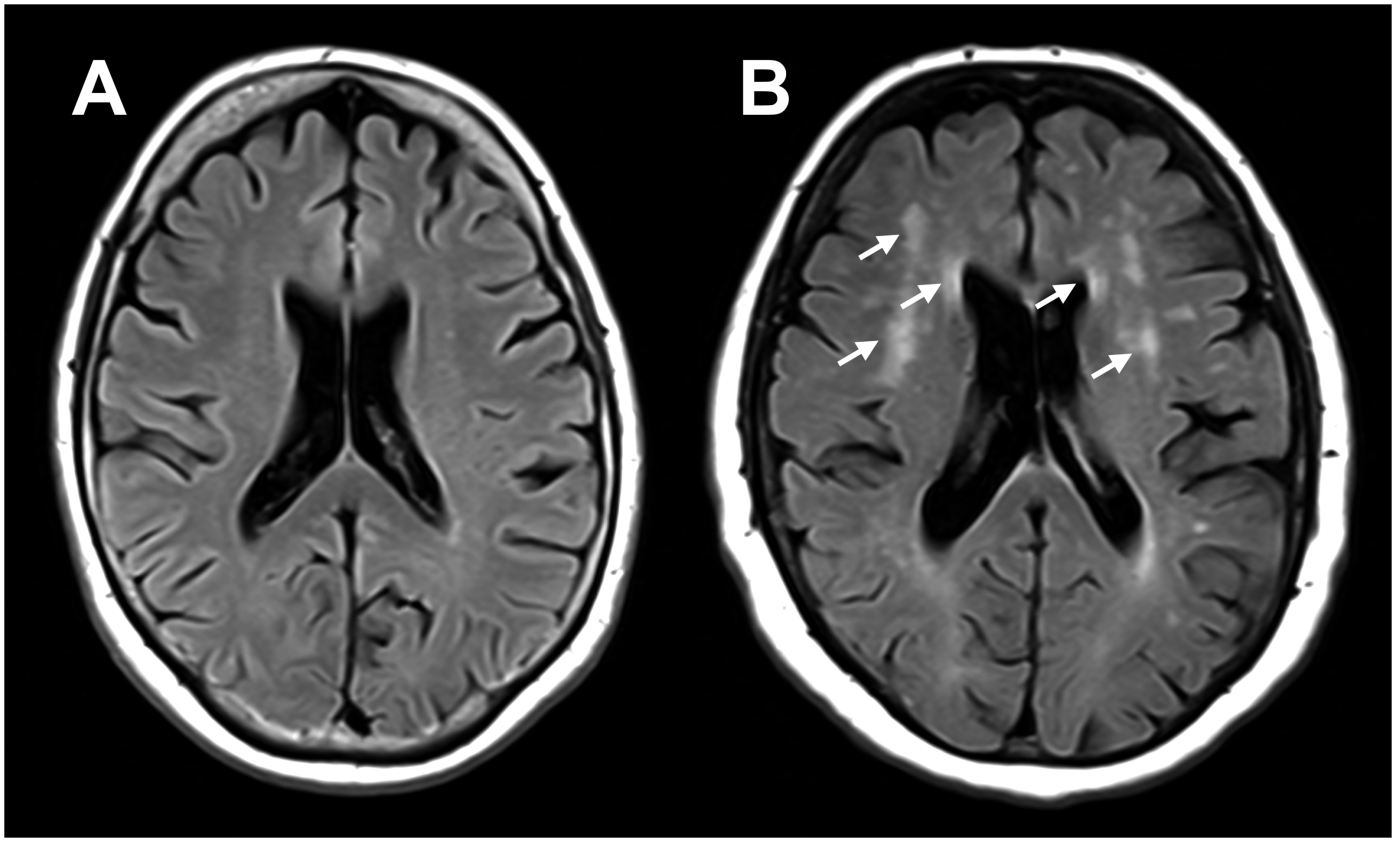
FLAIR images of two different representative subjects with different WMH load. Subject A represents lower WMH load (i.e. a WMH volume ≤ 0.5% of the intracranial volume that corresponded approximately to Fazekas grade 0–1) and subject B higher WMH load (i.e. a WMH volume ≥ 1% of the intracranial volume that corresponded approximately to Fazekas grade 2–3), with arrows indicating example regions with WMH.

Two subgroups with unequal WMH load, matched for age, male-to-female sex ratio, prevalence of cardiovascular disease, MMSE score, and ventricle volume, were selected from the group of cognitively healthy elderly (Table 1 and Fig 2). Lower WMH load was defined as having a WMH volume ≤ 0.5% of the total intracranial volume, which corresponded approximately to Fazekas grade 0–1, and higher WMH load as having a WMH volume ≥ 1%, which corresponded approximately to Fazekas grade 2–3 [12].

### Assessment of Aβ_42_/Aβ_40_ ratio in cerebrospinal fluid

The procedure and analysis of the CSF followed the Alzheimer’s Association Flow Chart for CSF biomarkers [49]. Lumbar CSF samples were collected at the three centers and analyzed according to a standardized protocol [49,50]. CSF Aβ_42_ and Aβ_40_ were analyzed by Euroimmun (EI) (EUROIMMUN AG, Lübeck, Germany).

### Statistical analysis

Statistical analysis was performed using the Statistical Package for Social Sciences (SPSS version 22, IBM Corp., Chicago, IL, USA).

A Mann-Whitney *U*-test for independent samples was performed to determine differences in age, male-to-female sex ratio, prevalence of cardiovascular disease, MMSE score, Aβ_42_/Aβ_40_ ratio, ventricle volume, and WMH volume between cognitively healthy elderly and prodromal AD subjects.

A two-tailed Student’s *t*-test for independent samples was performed to compare MD and FA in all eight white matter tracts between the two matched subgroups of cognitively healthy elderly with unequal WMH load. The effect size was based on differences in group means and described using Cohen’s *d*, which facilitates comparison of effect sizes between studies.

Two multivariate linear regression models were used to test if and to what degree the WMH load affected DTI estimates and their change in prodromal AD. In model 1, the dependent variable was the DTI estimate (MD or FA) for each tract (the dorsal and ventral cingulum, the SLF, and the CST) while the independent variables were having prodromal AD or not and ventricle volume. In model 2, WMH volume was added as an additional independent variable. Standardized β was calculated to describe the effect of the independent variables. In both models, variables tested using the Mann-Whitney *U*-test described above and with significant differences between the prodromal AD subjects and cognitively healthy elderly were also added as independent variables to be able to adjust the analyses for these. *R*^2^ was calculated to describe the proportion of the variance in the dependent variable that was predictable from the independent variable (i.e. how well the regression model explained the real data). Δ*R*^2^ was calculated to describe how *R*^2^ changes between model 1 and 2 (i.e. if adjusting the analysis for WMH volume improved the explanatory power of the analysis or not).

## Results

### Demographic analysis

In prodromal AD subjects, MMSE score was significantly higher (*p* = 1.23×10^−26^), Aβ_42_/Aβ_40_ ratio was significantly lower (*p* = 6.50×10^−70^), ventricular volume was significantly higher (*p* = 3.07×10^−3^), and WMH volume was significantly higher (*p* = 2.60×10^−4^) compared to cognitively healthy controls (Table 1). There were no significant differences in age, male-to-female sex ratio, or prevalence of cardiovascular disease between these groups.

### Effect of WMH on DTI estimates in cognitively healthy elderly

In cognitively healthy elderly with higher compared to lower WMH load (Table 2), MD was significantly elevated in the right SLF (0.81±0.06 vs. 0.75±0.03 μm^2^/ms, *p* = 2.47×10^−4^), the left SLF (0.79±0.06 vs. 0.73±0.03 μm^2^/ms, *p* = 1.83×10^−4^), and the right dorsal cingulum (0.76±0.04 vs. 0.74±0.03 μm^2^/ms, *p* = 0.04). Also, FA was significantly reduced in the right SLF (0.41±0.03 vs. 0.43±0.02, *p* = 1.61×10^−3^), the left SLF (0.41±0.03 vs. 0.44±0.02, *p* = 1.35×10^−3^), and the left dorsal cingulum (0.45±0.03 vs. 0.47±0.03, *p* = 0.03). Cohen’s *d* ranged from small (ventral cingulum, d < 0.3) to very high (SLF, d > 1.0).

**Table 2:** Comparison of MD and FA between the matched subgroups of cognitively healthy elderly with lower^a^ and higher^b^ WMH load, respectively, using a two-tailed Student’s *t*-test.

	MD (μm^2^/ms)			FA		
	Cognitively healthy elderly with lower^a^ WMH load (*n* = 21)	Cognitively healthy elderly with higher^b^ WMH load (*n* = 21)	Cohen’s *d*	Cognitively healthy elderly with lower^a^ WMH load (*n* = 21)	Cognitively healthy elderly with higher^b^ WMH load (*n* = 21)	Cohen’s *d*
DC right	0.74±0.03	0.76±0.04	0.63*	0.44±0.02	0.43±0.03	−0.52
DC left	0.74±0.04	0.77±0.04	0.61	0.47±0.03	0.45±0.03	−0.69*
VC right	0.71±0.05	0.71±0.07	0.05	0.39±0.02	0.39±0.02	−0.09
VC left	0.70±0.05	0.72±0.06	0.37	0.39±0.02	0.38±0.03	−0.42
SLF right	0.75±0.03	0.81±0.06	1.24*	0.43±0.02	0.41±0.03	−1.04*
SLF left	0.73±0.03	0.79±0.06	1.27*	0.44±0.02	0.41±0.03	−1.06*
CST right	0.69±0.05	0.71±0.06	0.39	0.53±0.03	0.52±0.03	−0.34
CST left	0.69±0.04	0.71±0.06	0.38	0.53±0.03	0.53±0.04	0.01

Values denote parameter estimates in the respective tract (mean±SD). Effect size is expressed as Cohen’s *d*. As expected, relatively large effect sizes are seen in regions where WMH are common (e.g. the SLF).

CST = corticospinal tract, DC = dorsal cingulum, FA = fractional anisotropy, MD = mean diffusivity, SLF = superior longitudinal fasciculus, VC = ventral cingulum, WMH = white matter hyperintensities.

^a^WMH volume ≤ 0.5% of the total intracranial volume, corresponding approximately to Fazekas score 0–1.

^b^WMH volume ≥ 1% of the total intracranial volume, corresponding approximately to Fazekas score 2–3.

* denote *p* < 0.05.

### Effect of WMH load in prodromal AD subjects compared to cognitively healthy elderly

Table 3 shows results from using regression model 1, where the dependent variable was MD or FA for each of the eight investigated tracts and the independent variables were having prodromal AD or not and ventricle volume. Tests showed that standardized β for having prodromal AD was significant for both MD and FA in the right SLF (β = 0.19 for MD and β = −0.13 for FA), the left SLF (β = 0.20 for MD and β = −0.16 for FA), the right dorsal cingulum (β = 0.16 for MD and β = −0.23 for FA), the left dorsal cingulum (β = 0.15 for MD and β = −0.20 for FA), the right ventral cingulum (β = 0.23 for MD and β = −0.18 for FA), and the left ventral cingulum (β = 0.27 for MD and β = −0.24 for FA). *R*^2^ ranged from 0.02 to 0.09. Thus, prodromal AD subjects had elevated MD and reduced FA in the right and left SLF and in the right and left dorsal and ventral cingulum compared to cognitively healthy elderly in the analysis not adjusted for WMH volume.

**Table 3:** Multivariate linear regression analyses of MD and FA in the dorsal and ventral cingulum, the SLF, and the CST in cognitively healthy elderly (*n* = 132) and prodromal AD subjects (*n* = 83) unadjusted (model 1) and adjusted (model 2) for WMH volume, respectively.

		Model 1 (unadjusted for WMH)		Model 2 (adjusted for WMH)		
		Standardized β (Prodromal AD)	*R*^*2*^	Standardized β (Prodromal AD)	Standardized β (WMH-volume)	Δ*R*^*2*^
MD	DC right	0.16*	0.03	0.12	0.21*	0.04*
	DC left	0.15*	0.02	0.09	0.26*	0.06*
	VC right	0.23*	0.05	0.23*	n.s.	n.s.
	VC left	0.27*	0.07	0.24*	n.s.	n.s.
	SLF right	0.19*	0.07	n.s.	0.74*	0.46*
	SLF left	0.20*	0.06	n.s.	0.74*	0.46*
	CST right	n.s.	0.02	n.s.	0.29*	0.07*
	CST left	n.s.	0.01	n.s.	0.24*	0.05*
FA	DC right	−0.23*	0.09	−0.16*	−0.28*	0.07*
	DC left	−0.20*	0.06	−0.16*	−0.17*	0.02*
	VC right	−0.18*	0.04	−0.18*	n.s.	n.s.
	VC left	−0.24*	0.06	−0.21*	n.s.	n.s.
	SLF right	−0.13*	0.03	n.s.	−0.61*	0.32*
	SLF left	−0.16*	0.04	n.s.	−0.61*	0.32*
	CST right	n.s.	0.04	n.s.	−0.33*	0.09*
	CST left	n.s.	0.09	n.s.	−0.21*	0.04*

In model 1, the dependent variable was MD or FA for each tract (the dorsal and ventral cingulum, the SLF, and the CST) and the independent variable were having prodromal AD or not. In model 2, WMH volume was added as an additional independent variable. Only standardized β for having aMCI or not and WMH volume are reported here. Results indicate that some of the differences in MD and FA between prodromal AD subjects and cognitively healthy elderly were largely driven by unequal WMH load between the groups rather than disease-specific alterations NAWM).

AD = Alzheimer's disease, CST = corticospinal tract, DC = dorsal cingulum, FA = fractional anisotropy, MD = mean diffusivity, NAWM = normal-appearing white matter, SLF = superior longitudinal fasciculus, VC = ventral cingulum, WMH = white matter hyperintensities.

* denote *p* < 0.05

n.s. denote non-significant.

In regression model 2 (see Table 3), where WMH volume was added as an additional independent variable, standardized β for having prodromal AD was significant for both MD and FA in the right ventral cingulum (β = 0.23 for MD and β = −0.18 for FA), the left ventral cingulum (β = 0.24 for MD and β = −0.21 for FA), and for FA in the right dorsal cingulum (β = −0.16) and left ventral cingulum (β = −0.16). In this model, standardized β for WMH volume was significant for both MD and FA in the right SLF (β = 0.74 for MD and β = −0.61 for FA), the left SLF (β = 0.74 for MD and −0.61 for FA), the right dorsal cingulum (β = 0.21 for MD and β = −0.28 for FA), the left dorsal cingulum (β = 0.26 for MD and −0.17 for FA), the right CST (β = 0.29 for MD and β = −0.33 for FA), and the left CST (β = 0.24 for MD and β = −0.21 for FA). Δ*R*^2^ was significant in all analyses where standardized β for WMH volume was significant and ranged from 0.02 to 0.46. Thus, in all investigated tracts except the ventral cingulum, the explanatory power of the model was improved (i.e. a higher *R*^2^ indicating better explained variance) when adjusting the analysis for WMH. Furthermore, in model 2, standardized β for having prodromal AD was no longer significant for MD or FA in neither the right nor the left SLF and no longer significant for MD in neither the right nor the left dorsal cingulum. That is, when adjusting the analysis for WMH there was no longer a significant difference in MD or FA in neither the right nor the left SLF and no longer a significant difference in MD in neither the right nor the left dorsal cingulum when comparing prodromal AD subjects to cognitively healthy elderly.

## Discussion

In the present study, we set out to investigate if unequal WMH load affects the comparison of DTI estimates between groups. First, we studied the effect of WMH in several major white matter tracts in cognitively healthy elderly. Second, we assessed the effect of WMH when comparing prodromal AD subjects to cognitively healthy elderly. We observed that the effect of WMH on MD and FA were in general large (Table 2). Moreover, when comparing MD and FA between prodromal AD subjects and cognitively healthy elderly, we observed that adjusting the analysis for WMH load improved the explanatory power as well as the outcome of the analysis. Our interpretation is that the effect of WMH on DTI estimates is comparable to that of and sometimes larger than disease-specific alterations in NAWM as reported by previous studies and that differences in DTI estimates were in some white matter tracts largely attributed to WMH load rather than being specific to prodromal AD (Table 3). These results are important because they suggest that unequal WMH load between two investigated groups could affect the result of a comparison of DTI estimates and may thus lead to an over- or underestimation of disease-specific alterations in NAWM. For example, a high WMH load in a control group may lead to false negative results. On the other hand, a high WMH load in a diseased group, in e.g. prodromal AD, may lead to false positive results. That is, if the aim of the study was to study disease-specific alterations in NAWM rather than WMH.

DTI estimates are affected by alterations in NAWM but also by WMH probably due to their similar composition on a microscopic level. Compared to normal white matter that largely consists of densely packed axons that restrict the diffusion of water molecules, alterations in NAWM are thought to represent axonal loss and demyelination leading to less restricted diffusion [51]. WMH, on the other hand, are thought to have a microstructure that consists of a varying degree of gliosis but also more severe axonal loss and demyelination that also leads to less restricted diffusion measured as elevated MD and reduced FA [11–13, 18]. Early alterations in NAWM, detectable only with DTI, are thought to be more disease-specific whereas WMH, detectable with DTI and T2-weigted MRI, are also encountered in cognitively healthy elderly [4–8, 14–17].

We found that the effect of WMH on MD and FA in cognitively healthy elderly was large and comparable to what has been reported previously for various pathological conditions. For example, effect sizes for MD in MCI are between 0.57 and 1.09, in AD between 0.95 and 1.04, in Parkinson’s disease between 0.78 and 2.20, and in schizophrenia between 0.78 and 1.24 [52−57]. Although the effect size is dependent on factors such as the cohort selection, the MRI scanner, the protocol used, and the method of analysis [58], it is worth noting that the significant effects we found on MD of high WMH load in matched subgroups of cognitively healthy elderly was between 0.63 and 1.27 for different tracts (Table 2). This is on par with several different pathological conditions (Table 4). Corresponding results were observed for FA. Adjusting for potentially unequal levels of WMH is thus warranted to disentangle effects of the WMH from more disease-specific alterations in NAWM.

**Table 4:** Effect size of group mean expressed as Cohen’s *d* and group size (*n*, patient group + control group) for WMH in the present study and various pathological conditions previously studied using DTI.

	Source	Condition	Region	Cohen’s *d*	*n*
MD	Present study	WMH	DC, VC, SLF, CST	0.63 to 1.27	21 + 21
	Rémy et al., 2015	MCI	Ventral cingulum, UF, Fornix	0.71 to 1.09	22 + 15
	Zhuang et al., 2013	MCI	Ventral cingulum, UF, Fornix	0.57 to 0.84	27 + 155
	Zhang et al., 2007	AD	Posterior and hippocampal cingulum	0.78 to 1.24	17 +18
	Gattellaro et al., 2009	PD	SLF, cingulum, genu of CC	0.78 to 2.20	10 + 10
FA	Present study	WMH	DC, VC, SLF, CST	−1.06 to −0.69	21 + 21
	Rémy et al., 2015	MCI	Ventral cingulum, UF, Fornix	−1.36 to −1.01	22 + 15
	Zhuang et al., 2013	MCI	Fornix	−0.74 to −0.61	27 + 155
	Stenset et al., 2011	MCI	Cingulum, genu of CC, forceps major	−0.94 to −0.62	12 +26
	Zhang et al., 2007	AD	Posterior and hippocampal cingulum and splenium of corpus callosum	−1.76 to −0.72	17 +18
	Gattellaro et al., 2009	PD	SLF, genu of CC	−1.50 to −0.97	10 + 10
	Wang et al., 2004	SZ	Anterior cingulum	−1.25 to −0.82	21 + 21

Only white matter tracts with significant differences in group mean are reported here.

AD = Alzheimer’s disease; FA = fractional anisotropy; MCI = mild cognitive impairment; MD = mean diffusivity; PD = Parkinson’s disease; SZ = schizophrenia, WMH = white matter hyperintensities.

The present study showed WMH to have a relatively large effect on DTI estimates even though we only took the WMH load into account, and not their exact location. This was probably due to a local effect of WMH on DTI estimates, large enough to affect the mean value of the white matter tract. The largest effect sizes were observed in the SLF and the dorsal cingulum, which are both at least partially located in regions where WMH are commonly observed (i.e. in periventricular and deep cortical regions) [5,17,30,31]. This suggests that there is a correlation between the effect of WMH on DTI estimates in a specific tract and its local WMH load. However, the current study was not designed to study this correlation and is therefore not able to neither confirm nor discard it.

When comparing DTI estimates in the eight investigated white matter tracts between prodromal AD subjects and cognitively healthy elderly using regression analysis we observed that MD was elevated and FA reduced, respectively, in prodromal AD subjects in the right and left dorsal and ventral cingulum and in the right and left SLF when the analysis was unadjusted for WMH load. These results are in accordance with most previous work although it has also been reported that FA can be unexpectedly elevated in the SLF of aMCI subjects due to a reduction of crossing fibers [5,59].

Adjusting for WMH load allowed us to separately test the effects of disease-specific alterations in NAWM and WMH in prodromal AD. When adjusting the analysis for WMH load we observed that the explanatory power as well as the outcome was improved for investigated white matter tracts at least partially located in regions where WMH are common (e.g. the SLF and the dorsal cingulum in the deep cortical regions of the frontal and parietal lobes) but not in regions were WMH are less common (e.g. the ventral cingulum in the temporal lobe) [17,30,31]. Furthermore, when adjusting the analysis for WMH load we still observed an elevated MD in the ventral cingulum and a reduced FA in the dorsal and ventral cingulum in prodromal AD subjects but no significant differences in MD in the SLF and dorsal cingulum or in FA in the SLF. This suggests that differences in DTI estimates in the SLF and partially in the dorsal cingulum between prodromal AD subjects and cognitively healthy elderly in the analysis unadjusted for WMH were largely driven by differences in WMH load between the two groups rather than disease-specific alterations in NAWM (Tables 1 and 3). This is not that surprising given that there was a group mean difference in WMH load between the prodromal AD subjects and the cognitively healthy elderly and that the largest effect size in group mean difference between the matched subgroups of cognitively healthy elderly with unequal WMH load were seen in the SLF and the dorsal cingulum. It is possible that these results extrapolate to studies of other conditions, and in particular in the elderly since the prevalence of WMH is known to increase with age [14–16].

We identified three limitations of the present study. First, the exact etiology of WMH in the study population was unknown. Even though we matched and adjusted the analyses for the influence of cardiovascular disease, WMH could in some subjects be an expression of other incipient disease (e.g. multiple sclerosis) that DTI could be sensitive to. However, we made an effort to minimize this potential confounding effect when designing the inclusion and exclusion criteria. Second, since WMH have a different microscopic structure compared to NAWM, WMH may interfere with fiber tracking and thus the presence of WMH in the investigated groups may potentially have biased the results. Even though this is a theoretical limitation, visual inspection of the generated tractographies awoke no suspicions that this was the case in this study. Third, we did not correct for multiple comparisons due to the explorative nature of the study. This means that we preferred the risk of accepting false positive differences over the risk of discarding true positive differences.

## Conclusion

The effect of unequal WMH load in cognitively healthy elderly on the DTI estimates MD and FA was generally large and comparable to previous reports from using DTI in various pathological conditions such as neurodegenerative disease. Adjusting the statistical analysis for WMH load when comparing MD and FA between prodromal AD subjects and cognitively healthy elderly improved the explanatory power as well as the outcome of the analysis indicating that some differences in MD and FA were largely driven by unequal WMH load between the groups rather than disease-specific alterations in NAWM. This suggests that the effect of differences in WMH load between groups is large enough to affect the results of a statistical analysis of DTI estimates and that this should be taken into consideration in the design and interpretation of DTI studies. If the purpose of a DTI study is to compare disease-specific alterations in NAWM rather than WMH between groups with unequal WMH load this can be achieved by adjusting the statistical analysis for WMH load.
